# Supplementary material for: Predictive usefulness of RT-PCR testing in different patterns of Covid-19 symptomatology: analysis of a French cohort of 12,810 outpatients
Source: Sci Rep. 2021 Oct 27;11:21233. doi: 10.1038/s41598-021-99991-6 (PMC8551264; doi:10.1038/s41598-021-99991-6)
Supplement: Supplementary file 1 — Supplementary Information 1. [file 41598_2021_99991_MOESM1_ESM.pdf]

Figure S1

Questionnaire COVIDOM

(partial relevant extract)

1. Principaux antécédents	1. Medical history
Quel est votre poids habituel ? kg<br>Quelle est votre taille ? cm	What is your usual weight ? kg<br>What is your height ? cm
Souffrez-vous de diabète ? oui/non<br>Souffrez-vous d'hypertension ? oui/non<br>Souffrez-vous de BPCO ou d'emphysème ? oui/non<br>Souffrez-vous d'asthme ? oui/non<br>Souffrez-vous d'infarctus ou d'angine de poitrine ? oui/non<br>Souffrez-vous d'insuffisance cardiaque ? oui/non<br>Souffrez-vous d'un cancer en cours de traitement ? oui/non<br>Souffrez-vous d'insuffisance rénale ? oui/non<br>Souffrez-vous d'une autre maladie chronique ? oui/non ; Précisez	Do you have diabetes ? yes / no<br>Do you have hypertension ? yes / no<br>Do you have COPD or emphysema ? yes / no<br>Do you have asthma ? yes / no<br>Do you have heart infarct or angina ? yes / no<br>Do you have heart failure ? yes / no<br>Do you have cancer with ongoing treatment ? yes / no<br>Do you have chronic kidney disease ? yes / no<br>Do you have other chronical diseases ? yes/no; Precise
Êtes-vous fumeur ? oui/non	Do you smoke ? yes / no
2. Symptômes	2. Symptoms
Avez-vous, au cours de votre maladie, présenté l'un ou les symptômes suivants ? cases à cocher	During your disease, did you have any of the following signs ?
Fatigue	Tiredness
Fièvre (température >38°5C)	Fever
Frissons	Shivers
Toux	Cough
Gêne respiratoire (essoufflement)	Breathlessness
Douleurs musculaires (myalgies)	Aches in the muscles
Sensation d'oppression thoracique de plusieurs minutes	Chest oppression lasting several minutes
Douleurs thoraciques	Chest pain
Diarrhées	Diarrhea
Nausées, vomissements	Nausea, vomiting
Perte de l'odorat	Loss of smell
Perte du goût	Loss of appetite
Manque d'appétit	Lack of appetite
Eruption cutanée, urticaire	Rash, hives
Engelures	Frostbites
Conjonctivite	Conjunctivitis
Autres (Précisez)	Other symptoms (Precise)

3. Examens complémentaires<br><br>Avez-vous eu un test de l'infection à Covid-19 ? oui/non<br>Etait-il positif / négatif ?<br><br>Avez-vous passé un scanner des poumons dans le cadre de cet épisode infectieux ? oui/non<br><br>Avez-vous passé une radiographie des poumons dans le cadre de cet épisode infectieux ? oui/non	3. Investigations<br><br>Did you benefit from a Covid-19 test ? yes/no<br><br>Was it positive / negative for Covid ?<br><br>Did you have a lung scan as part of this infectious episode? yes/no<br><br>Did you have a chest X-ray as part of this infectious episode? yes/no

Figure S2

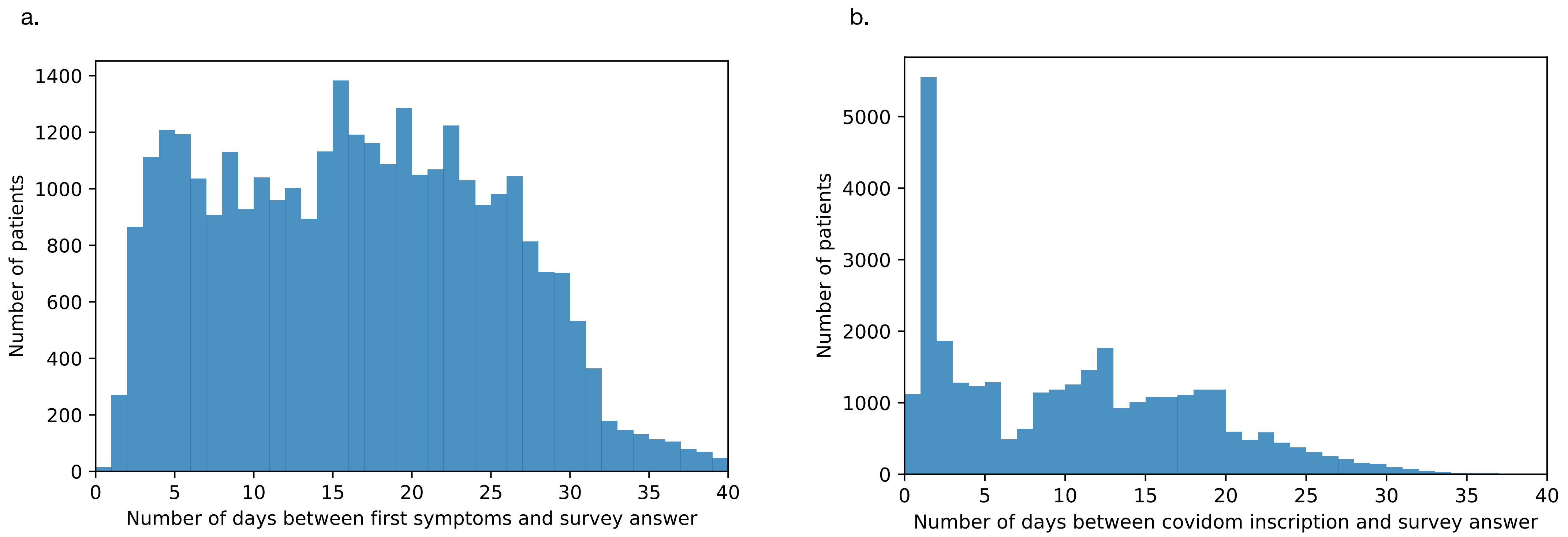

a.
Number of patients
Number of days between first symptoms and survey answer
b.
Number of patients
Number of days between covidom inscription and survey answer

Figure S3

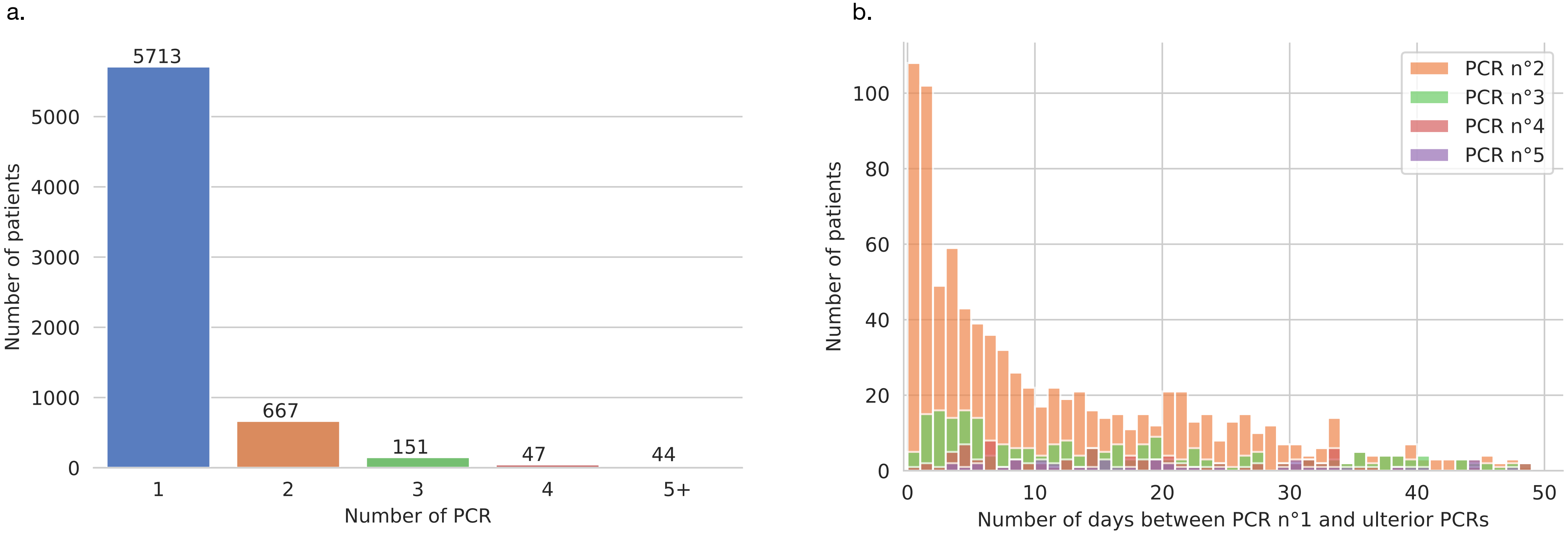
a.
5713
667
151
47
44
Number of patients
0
1000
2000
3000
4000
5000
1
2
3
4
5+
Number of PCR
b.
PCR n°2
PCR n°3
PCR n°4
PCR n°5
Number of patients
0
20
40
60
80
100
0
10
20
30
40
50
Number of days between PCR n°1 and ulterior PCRs

Figure S4

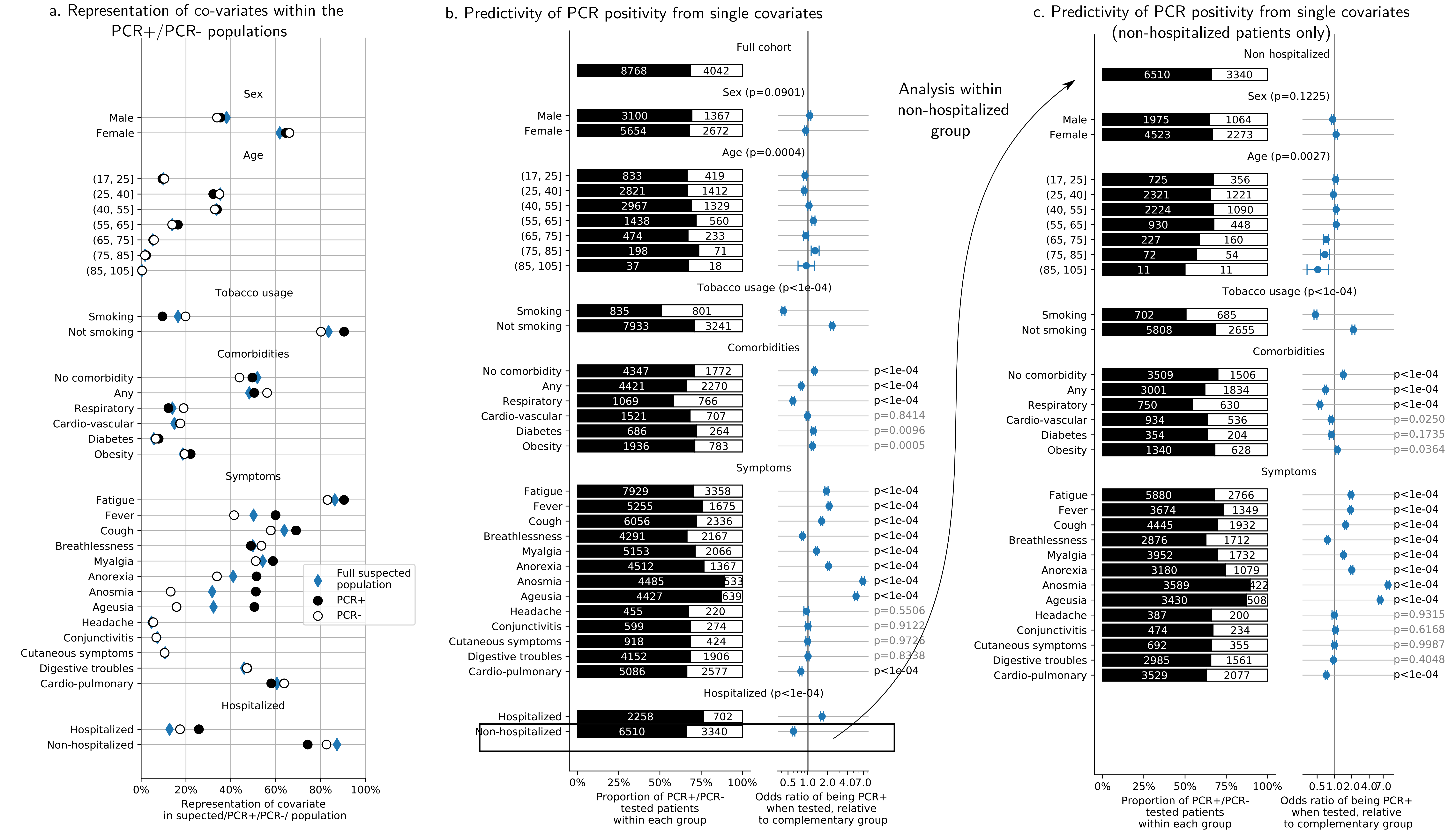
a. Representation of co-variates within the PCR+/PCR- populations
Sex
Male
Female
Age
(17, 25]
(25, 40]
(40, 55]
(55, 65]
(65, 75]
(75, 85]
(85, 105]
Tobacco usage
Smoking
Not smoking
Comorbidities
No comorbidity
Any
Respiratory
Cardio-vascular
Diabetes
Obesity
Symptoms
Fatigue
Fever
Cough
Breathlessness
Myalgia
Anorexia
Anosmia
Ageusia
Headache
Conjunctivitis
Cutaneous symptoms
Digestive troubles
Cardio-pulmonary
Hospitalized
Hospitalized
Non-hospitalized
Full suspected population
PCR+
PCR-
0%
20%
40%
60%
80%
100%
Representation of covariate in supected/PCR+/PCR-/ population
b. Predictivity of PCR positivity from single covariates
Full cohort
8768 4042
Sex (p=0.0901)
Male 3100 1367
Female 5654 2672
Age (p=0.0004)
(17, 25] 833 419
(25, 40] 2821 1412
(40, 55] 2967 1329
(55, 65] 1438 560
(65, 75] 474 233
(75, 85] 198 71
(85, 105] 37 18
Tobacco usage (p<1e-04)
Smoking 835 801
Not smoking 7933 3241
Comorbidities
No comorbidity 4347 1772 p<1e-04
Any 4421 2270 p<1e-04
Respiratory 1069 766 p<1e-04
Cardio-vascular 1521 707 p=0.8414
Diabetes 686 264 p=0.0096
Obesity 1936 783 p=0.0005
Symptoms
Fatigue 7929 3358 p<1e-04
Fever 5255 1675 p<1e-04
Cough 6056 2336 p<1e-04
Breathlessness 4291 2167 p<1e-04
Myalgia 5153 2066 p<1e-04
Anorexia 4512 1367 p<1e-04
Anosmia 4485 533 p<1e-04
Ageusia 4427 639 p<1e-04
Headache 455 220 p=0.5506
Conjunctivitis 599 274 p=0.9122
Cutaneous symptoms 918 424 p=0.9726
Digestive troubles 4152 1906 p=0.8338
Cardio-pulmonary 5086 2577 p<1e-04
Hospitalized (p<1e-04)
Hospitalized 2258 702
Non-hospitalized 6510 3340
0% 25% 50% 75% 100%
Proportion of PCR+/PCR- tested patients within each group
0.5 1.0 2.0 4.0 7.0
Odds ratio of being PCR+ when tested, relative to complementary group
Analysis within non-hospitalized group
c. Predictivity of PCR positivity from single covariates (non-hospitalized patients only)
Non hospitalized
6510 3340
Sex (p=0.1225)
Male 1975 1064
Female 4523 2273
Age (p=0.0027)
(17, 25] 725 356
(25, 40] 2321 1221
(40, 55] 2224 1090
(55, 65] 930 448
(65, 75] 227 160
(75, 85] 72 54
(85, 105] 11 11
Tobacco usage (p<1e-04)
Smoking 702 685
Not smoking 5808 2655
Comorbidities
No comorbidity 3509 1506 p<1e-04
Any 3001 1834 p<1e-04
Respiratory 750 630 p<1e-04
Cardio-vascular 934 536 p=0.0250
Diabetes 354 204 p=0.1735
Obesity 1340 628 p=0.0364
Symptoms
Fatigue 5880 2766 p<1e-04
Fever 3674 1349 p<1e-04
Cough 4445 1932 p<1e-04
Breathlessness 2876 1712 p<1e-04
Myalgia 3952 1732 p<1e-04
Anorexia 3180 1079 p<1e-04
Anosmia 3589 422 p<1e-04
Ageusia 3430 508 p<1e-04
Headache 387 200 p=0.9315
Conjunctivitis 474 234 p=0.6168
Cutaneous symptoms 692 355 p=0.9987
Digestive troubles 2985 1561 p=0.4048
Cardio-pulmonary 3529 2077 p<1e-04
0% 25% 50% 75% 100%
Proportion of PCR+/PCR- tested patients within each group
0.5 1.0 2.0 4.0 7.0
Odds ratio of being PCR+ when tested, relative to complementary group

Figure S5

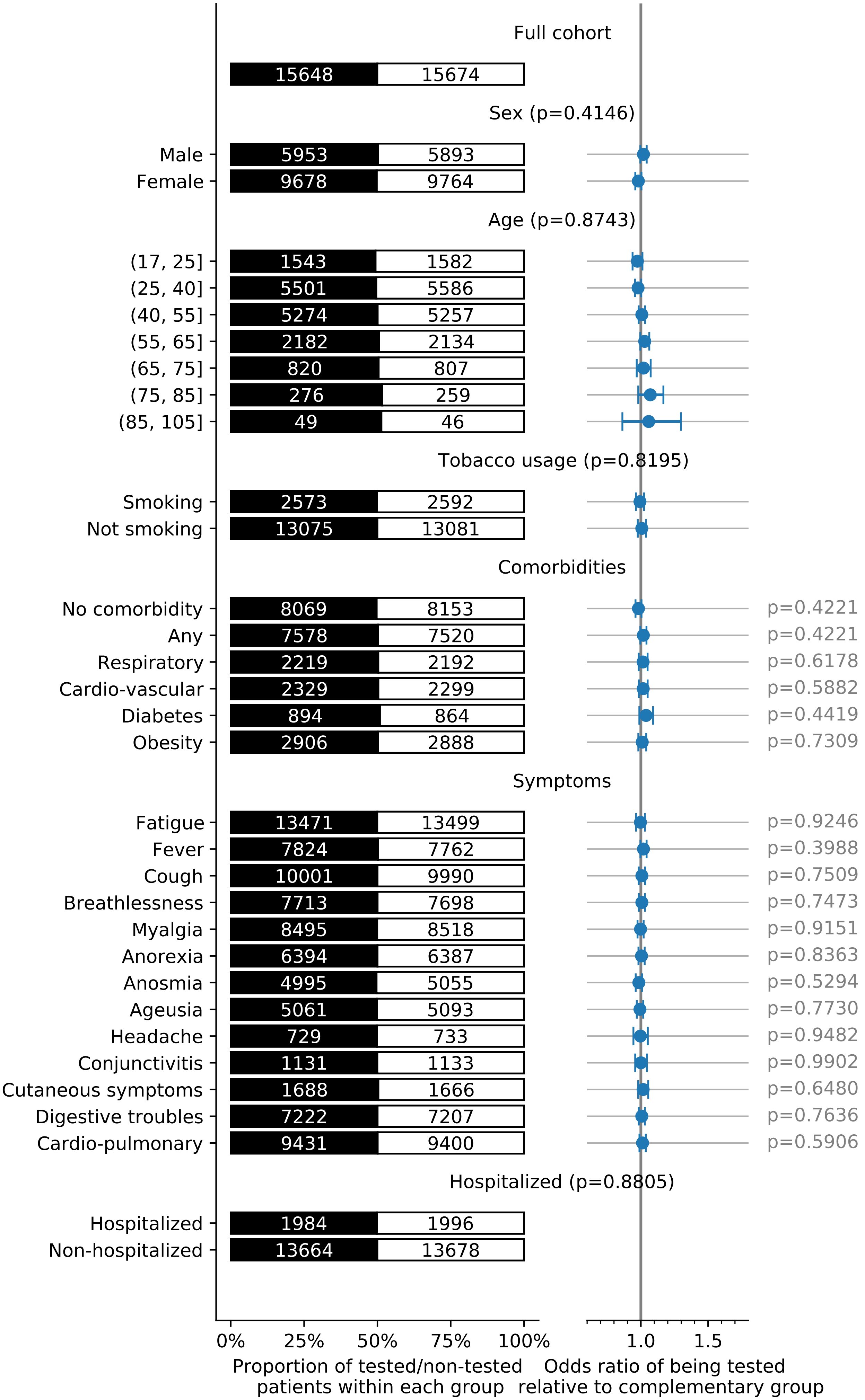

Full cohort
15648
15674
Sex (p=0.4146)
Male
5953
5893
Female
9678
9764
Age (p=0.8743)
(17, 25]
1543
1582
(25, 40]
5501
5586
(40, 55]
5274
5257
(55, 65]
2182
2134
(65, 75]
820
807
(75, 85]
276
259
(85, 105]
49
46
Tobacco usage (p=0.8195)
Smoking
2573
2592
Not smoking
13075
13081
Comorbidities
No comorbidity
8069
8153
p=0.4221
Any
7578
7520
p=0.4221
Respiratory
2219
2192
p=0.6178
Cardio-vascular
2329
2299
p=0.5882
Diabetes
894
864
p=0.4419
Obesity
2906
2888
p=0.7309
Symptoms
Fatigue
13471
13499
p=0.9246
Fever
7824
7762
p=0.3988
Cough
10001
9990
p=0.7509
Breathlessness
7713
7698
p=0.7473
Myalgia
8495
8518
p=0.9151
Anorexia
6394
6387
p=0.8363
Anosmia
4995
5055
p=0.5294
Ageusia
5061
5093
p=0.7730
Headache
729
733
p=0.9482
Conjunctivitis
1131
1133
p=0.9902
Cutaneous symptoms
1688
1666
p=0.6480
Digestive troubles
7222
7207
p=0.7636
Cardio-pulmonary
9431
9400
p=0.5906
Hospitalized (p=0.8805)
Hospitalized
1984
1996
Non-hospitalized
13664
13678
0%
25%
50%
75%
100%
1.0
1.5
Proportion of tested/non-tested patients within each group
Odds ratio of being tested relative to complementary group

Figure S6

Full decision tree to predict PCR on held-out patients

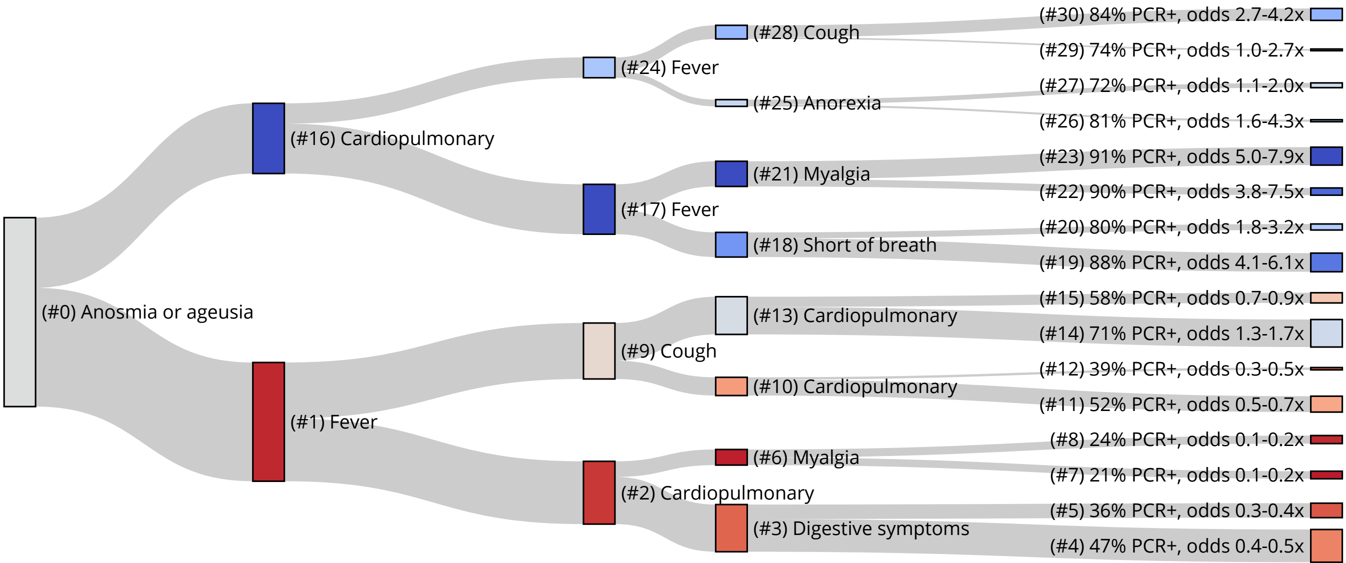

Figure S7

Decision tree to predict PCR on the entire tested cohort

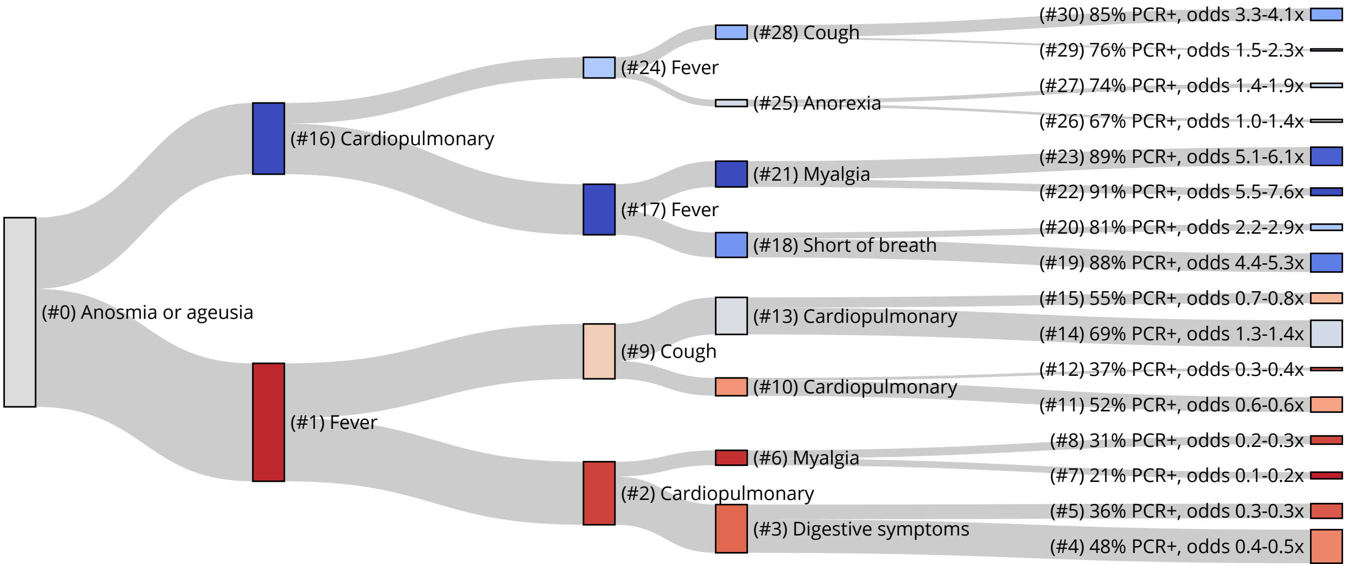

Figure S8

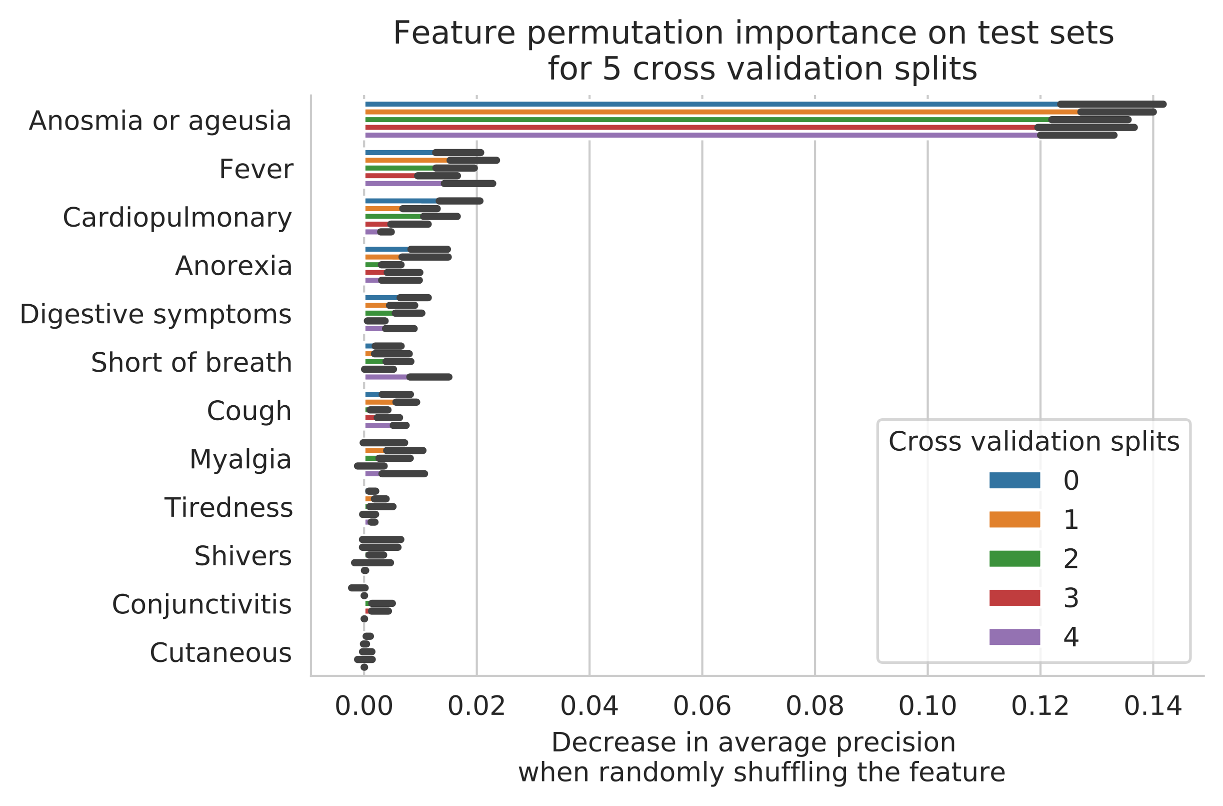

Feature permutation importance on test sets
for 5 cross validation splits
Anosmia or ageusia
Fever
Cardiopulmonary
Anorexia
Digestive symptoms
Short of breath
Cough
Myalgia
Tiredness
Shivers
Conjunctivitis
Cutaneous
0.00
0.02
0.04
0.06
0.08
0.10
0.12
0.14
Decrease in average precision
when randomly shuffling the feature
Cross validation splits
0
1
2
3
4

Figure S9

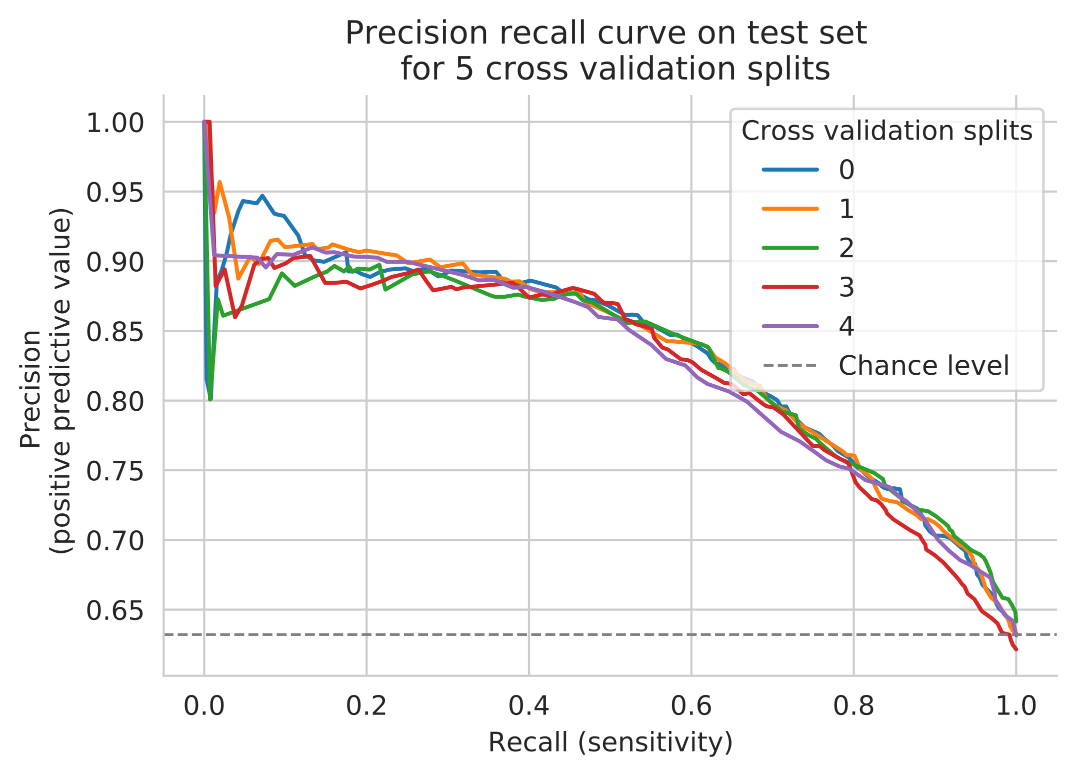
Precision recall curve on test set
for 5 cross validation splits
Precision
(positive predictive value)
Recall (sensitivity)
1.00
0.95
0.90
0.85
0.80
0.75
0.70
0.65
0.0
0.2
0.4
0.6
0.8
1.0
Cross validation splits
0
1
2
3
4
Chance level

Figure S10

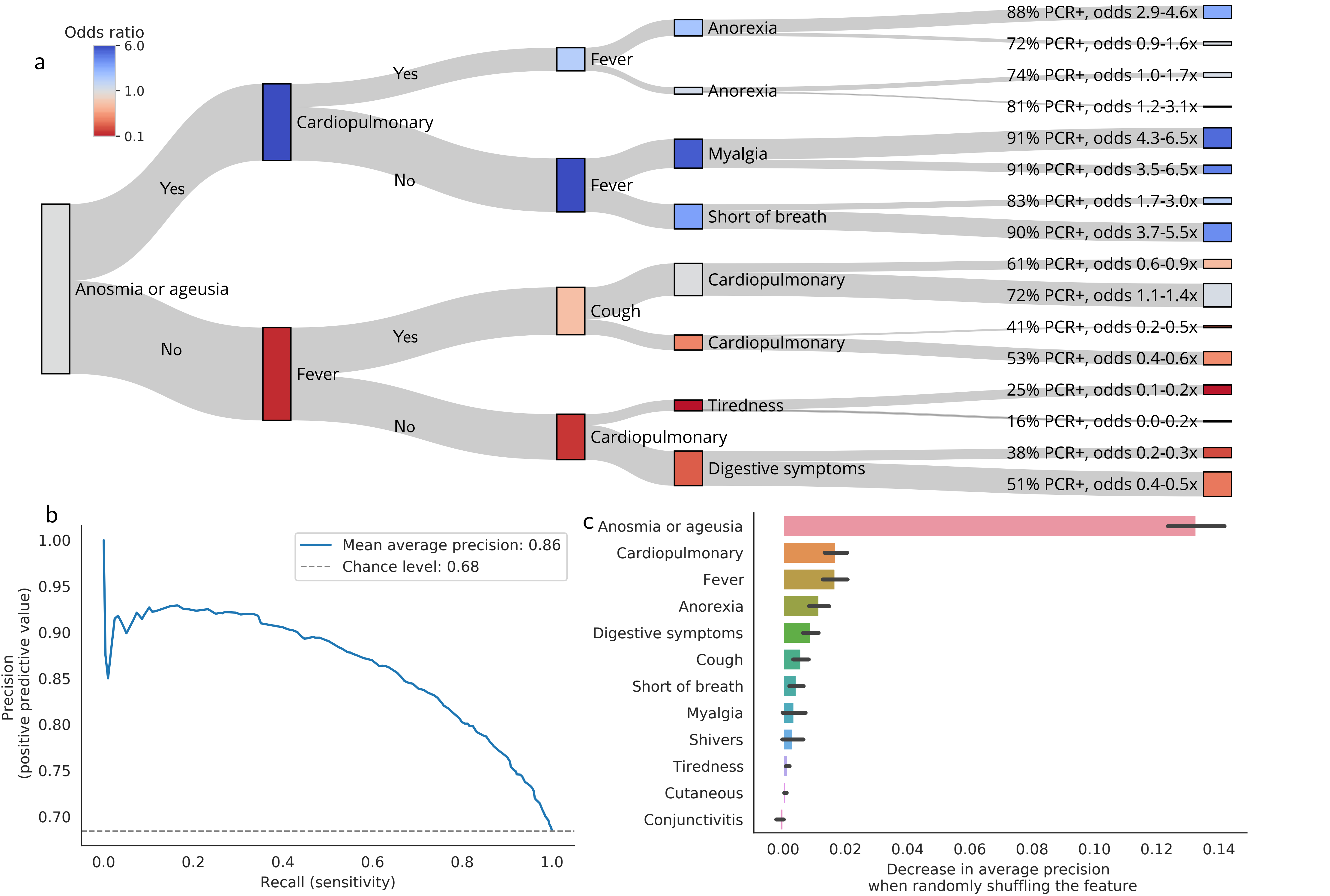
a
Odds ratio
6.0
1.0
0.1
Anosmia or ageusia
Yes
No
Cardiopulmonary
Fever
Yes
No
Yes
No
Fever
Fever
Cough
Cardiopulmonary
Anorexia
Anorexia
Myalgia
Short of breath
Cardiopulmonary
Cardiopulmonary
Tiredness
Digestive symptoms
88% PCR+, odds 2.9-4.6x
72% PCR+, odds 0.9-1.6x
74% PCR+, odds 1.0-1.7x
81% PCR+, odds 1.2-3.1x
91% PCR+, odds 4.3-6.5x
91% PCR+, odds 3.5-6.5x
83% PCR+, odds 1.7-3.0x
90% PCR+, odds 3.7-5.5x
61% PCR+, odds 0.6-0.9x
72% PCR+, odds 1.1-1.4x
41% PCR+, odds 0.2-0.5x
53% PCR+, odds 0.4-0.6x
25% PCR+, odds 0.1-0.2x
16% PCR+, odds 0.0-0.2x
38% PCR+, odds 0.2-0.3x
51% PCR+, odds 0.4-0.5x
b
Precision
(positive predictive value)
Recall (sensitivity)
Mean average precision: 0.86
Chance level: 0.68
1.00
0.95
0.90
0.85
0.80
0.75
0.70
0.0
0.2
0.4
0.6
0.8
1.0
c
Anosmia or ageusia
Cardiopulmonary
Fever
Anorexia
Digestive symptoms
Cough
Short of breath
Myalgia
Shivers
Tiredness
Cutaneous
Conjunctivitis
0.00
0.02
0.04
0.06
0.08
0.10
0.12
0.14
Decrease in average precision
when randomly shuffling the feature

Figure S11

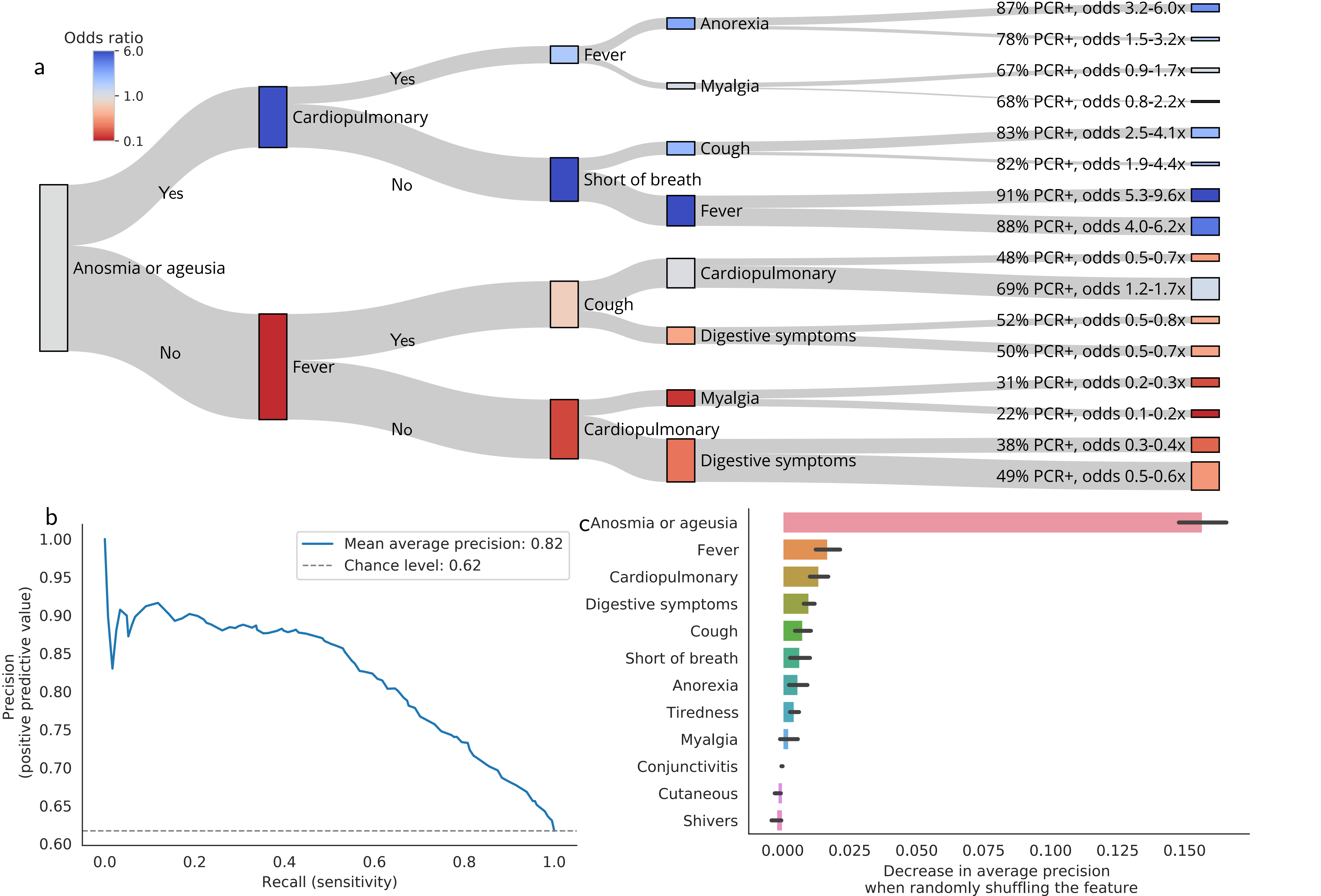
a
Odds ratio
6.0
1.0
0.1
Anosmia or ageusia
Yes
No
Cardiopulmonary
Fever
Yes
No
Yes
No
Fever
Short of breath
Cough
Cardiopulmonary
Anorexia
Myalgia
Cough
Fever
Cardiopulmonary
Digestive symptoms
Myalgia
Digestive symptoms
87% PCR+, odds 3.2-6.0x
78% PCR+, odds 1.5-3.2x
67% PCR+, odds 0.9-1.7x
68% PCR+, odds 0.8-2.2x
83% PCR+, odds 2.5-4.1x
82% PCR+, odds 1.9-4.4x
91% PCR+, odds 5.3-9.6x
88% PCR+, odds 4.0-6.2x
48% PCR+, odds 0.5-0.7x
69% PCR+, odds 1.2-1.7x
52% PCR+, odds 0.5-0.8x
50% PCR+, odds 0.5-0.7x
31% PCR+, odds 0.2-0.3x
22% PCR+, odds 0.1-0.2x
38% PCR+, odds 0.3-0.4x
49% PCR+, odds 0.5-0.6x
b
Mean average precision: 0.82
Chance level: 0.62
Precision (positive predictive value)
1.00
0.95
0.90
0.85
0.80
0.75
0.70
0.65
0.60
0.0
0.2
0.4
0.6
0.8
1.0
Recall (sensitivity)
c
Anosmia or ageusia
Fever
Cardiopulmonary
Digestive symptoms
Cough
Short of breath
Anorexia
Tiredness
Myalgia
Conjunctivitis
Cutaneous
Shivers
0.000
0.025
0.050
0.075
0.100
0.125
0.150
Decrease in average precision when randomly shuffling the feature
