# Supplementary material for: Predictive usefulness of RT-PCR testing in different patterns of Covid-19 symptomatology: analysis of a French cohort of 12,810 outpatients
Source: Sci Rep. 2021 Oct 27;11:21233. doi: 10.1038/s41598-021-99991-6 (PMC8551264; doi:10.1038/s41598-021-99991-6)
Supplement: Supplementary file 2 — Supplementary Information 2. [file 41598_2021_99991_MOESM2_ESM.docx]

Predictive usefulness of RT-PCR testing in different patterns of Covid-19 symptomatology

Analysis of a French cohort of 12,810 outpatients

On behalf of the AP-HP/Universities/Inserm COVID-19 research collaboration

**Authors:**

# AP-HP / Universities / Inserm COVID-19 research collaboration members

**Writing & data science:**

APRA Caroline*✦^1,2^, CAUCHETEUX Charlotte✦^3^, MENSCH Arthur✦^4^, MANSOUR Jenny ^5^, BERNAUX Mélodie ^6^, DECHARTRES Agnès ^7,8^, DEBUC Erwan ^9,10^, LESCURE Xavier ^11,12^, DINH Aurélien ^13^, PARIS Nicolas ^16^, GRAMFORT Alexandre ^3^, YORDANOV Youri ^7,9^, JOURDAIN Patrick ^14,15^

✦ Those authors contributed equally to this work

**Affiliations:**

^1^ Sorbonne Université, Paris, France

^2^ Sorbonne Université, AP-HP, Neurosurgery Department, Pitié-Salpêtrière Hospital, Paris, France

^3^ Université Paris-Saclay, INRIA, CEA, Palaiseau, 91120, France

^4^ Ecole Normale Supérieure, PSL University, CNRS, Département de Mathématiques et Applications, Paris, France

^5^ Ecole Polytechnique, Paris, France

^6^ AP-HP, Direction de la stratégie et de la transformation, Paris, France

^7^ Sorbonne Université, INSERM, Institut Pierre Louis d'Epidémiologie et de Santé Publique, UMR-S 1136, Paris, France

^8^ Sorbonne Université, AP-HP, Pitié-Salpêtrière Hospital, Département de Santé Publique, Centre de Pharmaco Épidémiologie de l’AP-HP (Cephepi), Paris, France

^9^ Sorbonne Université, AP-HP, Saint Antoine Hospital, Service d'Accueil des Urgences, Paris, France

^10^ Assistance Publique-Hôpitaux de Paris, Paris, France

^11^ Department of Infectious and Tropical Diseases , APHP, Bichat-Claude Bernard University Hospital, Paris, France

^12^ Infections Antimicrobials Modelling Evolution (IAME) UMR 1137, University of Paris, Paris, France

^13^ Infectious disease department, R. Poincaré University Hospital, Garches, APHP, Paris Saclay University, Paris, France

^14^ DMU COREVE, GHU Paris Saclay, APHP, Paris, France

^15^ INSERM UMR S 999, IHU TORINO (thorax innovation), Paris, France

^16^ Entrepôt des Données de Santé, Assistance Publique-Hôpitaux de Paris, Paris, France

**Scientific committee:** AIME-EUSEBI Amélie, APRA Caroline, BLEIBTREU Alexandre, DEBUC Erwan, DECHARTRES Agnès, DECONINCK Laurène, DINH Aurélien, JOURDAIN Patrick, KATLAMA Christine, LEBEL Josselin, LESCURE François-Xavier, YORDANOV Youri

**Covidom regional center steering committee**: ARTIGOU Yves, BANZET Amélie, BOUCHERON Elodie, BOUDIER Christiane, BUZENAC Edouard, CHAPRON Marie-Claire, CHEKAOUI Dalhia, DE BASTARD Laurent, DEBUC Erwan, DINH Aurélien, GRENIER Alexandre, HAAS Pierre-Etienne, HODY Julien, JARRAYA Michèle, JOURDAIN Patrick, LACAILLE Louis, LE GUERN Aurélie, LECLERT Jeremy, MALE Fanny, MARCHAND-ARVIER Jerôme, MARTIN-BLONDET Emmanuel, NASSOUR Apolinne, OURAHOU Oussama, PENN Thomas, RIBARDIERE Ambre, ROBIN Nicolas, ROUGE Camille, SCHMIDT Nicolas, VILLIE Pascaline

* Corresponding author:

Caroline APRA

caroline.apra@aphp.fr

0033-142163208

Neurosurgical Department

Pitié Salpêtrière Hospital

Supplementary data

## Supplementary methods

## Estimating the decision tree

We select the best hyper-parameters among the followings: maximum depth of the tree (varying between 1 and no limitation), minimum number of samples in a leaf (between 50 and 1000), minimum number of samples when splitting (between 5 and 1000), minimum decrease in impurity when splitting (between 1e-3 and 1e-8). We used Gini as homogeneity criteria, and a stratified 5-folds cross-validation scheme on the train set. This results in 4224 different models with average precision varying between 0.83 and 0.89, the baseline performance being 0,75. The best decision tree (maximum depth of 8, minimum samples in a leaf of 100, minimum decrease in impurity of 1e-7, minimum samples in a split of 5) is then estimated on the whole training set and evaluated on the left-out data (20% of the dataset). By keeping such a held-out patient group for evaluation, the performance reported is statistically unbiased.

To evaluate the relevance of each feature at predicting RT-PCR positivity, we compute the variable importance on the test set for 50 random permutations (22). The variable importance, commonly referred to as “permutation importance”, measures the decrease in a model performance (here, average precision) when a single feature is randomly permuted between patients. It boils down to hiding the variable from the model while keeping the same marginal distribution unchanged.

To evaluate the variability of the predictions, we repeat the evaluation procedure (hyper-parameter selection using cross-validation on the train set and tree estimation) for 5 different train/test splits.

Table S1: data associated with figure 2, effects of patient characteristics on RT-PCR results

## Figures / Tables / Legends

## Figure S1: **Extract for the questionnaire sent to the patients included in the COVIDOM web-application.** Originally in French, translated for the purpose of this publication.

Figure S2: **Time repartition of the answers to the survey.**

Data was available for the patients with known start symptom date (n=31,242). There was a median of 16 days (IQ9-23) after the first symptoms and 10 days (IQ2-16) after the inclusion in COVIDOM when the patients filled up the survey. a) Number of days between COVIDOM inscription and the answer. b) Number of days between declaration of the first symptoms and the answer.

Figure S3: **RT-PCR and time**

a. Delay between successive RT-PCR for a subgroup of patients. Data was available for the patients included in the EDS database with a known number of RT-PCR tests (n=6,622). The average number of RT-PCR tests was 1.2+/-0.6 and the median time between RT-PCR1 and RT-PCR2 was 7 days (only for patients with known RT-PCR date, n=6541).

b. Effect of time on RT-PCR result. Data was available for the patients included in the EDS database, with known start symptom date, first RT-PCR validation date and result (n=5,761). For patients who were tested more than one time, only the first RT-PCR is taken into account. In white, the number of patients whose first RT-PCR was validated X days after the first symptoms, and positive.

Figure S4: **Effect of patient characteristics on RT-PCR results, without propensity score**

Result of a univariate analysis of factors explaining RT-PCR results, without the propensity-score weighting. Results are similar with those of Figure 2b).

Figure S5: **Access to RT-PCR testing using pseudo-counts**

Each patient is weighted using a testing-propensity score based on a multivariate logistic model (see methods). Propensity score weighting properly removes the testing-propensity confound: for each characteristic, approximately half of the weighted population has been tested, and half has not. Completes Figure 1.

## Action of the decision tree on the evaluation and full dataset

Figure S6: **Decision path for the patients of the evaluation dataset** (20% of the dataset).

Complete numerical data from figures 3.a). Each split corresponds to the presence (up) or absence of the symptom (down). Each leaf is identified by # and the number of patients, RT-PCR results and odds ratios are detailed in the table (b) Numerical data TableS2.

Table S2: data associated with the decision path, for the patients of the evaluation dataset (20% of all patients)

Figure S7: **Decision path for all samples** (100% of the dataset). Cf Fig.S6 for reading.

This tree confirms the same discriminant features as those found in the partial dataset for the first three splits.

(b) Numerical data TableS3.

Table S3: data associated with the decision path, for all samples

## Stability of the estimation procedure across test/train splits

Figure S8: **Stability of the tree performance in 5-fold cross validation - Performance of the decision tree on the 5 test sets.** We repeat the training procedure and hyper-parameter tuning on 5 cross-validated stratified folds with 20% left-out data. Here are the five performance curves of the 5 resulting decision trees. Each colour corresponds to one split.

Figure S9: **Stability of the tree performance in 5-fold cross validation -** **Permutation features importance on the 5 test sets.** We compute the feature permutation importance on each of the five test sets for 50 permutations. Each colour corresponds to one fold. The error bars correspond to the permutation importance variability through permutations.

## Varying decision tree estimation choices

Fig.S10 : **Tree learned from samples without propensity-score weighting**

Results are similar with results from Figure 3, showing the robustness of the tree classification to weighting strategies. In particular, the first splits used in Figure 4 are unchanged with or without propensity-score weighting

Fig.S11 : **Tree learned from samples on the ambulatory population only**

Results are similar with results from Figure 3. In particular, the first splits used in Figure 4 are unchanged with or without propensity-score weighting.
